# How to Control for Gestational Age: Olsen and Fei Respond

**DOI:** 10.1289/ehp.11105R

**Published:** 2008-07

**Authors:** Jørn Olsen, Chunyuan Fei

**Affiliations:** Department of Epidemiology University of California, Los Angeles Los Angeles, California E-mail: jo@ucla.edu

As described by Slama et al., it is not a simple matter to adjust for gestational age when analyzing birth weights. Any estimate of gestational age is prone to misclassification, whether it is based on ultrasound or last menstrual period (LMP). Ultrasound measures are based on the assumption of uniform early fetal growth or at least that the exposure under study has no impact on early fetal growth. This assumption is probably not always correct, as first demonstrated by Henriksen et al. (1995). LMP estimates are prone to large random measurement errors that may become non-random if the exposures under study affects menstrual bleeding patterns.

Although these problems are part of textbook knowledge (Olsen and Basso 2007), their impact appears to be limited in our experience. In our study (Fei et al. 2007), the analyses based primarily on LMP estimates provided a regression coefficient of −10.35 [95% confidential interval (CI), −20.6 to −0.15] between perfluorooctanoate and birth weight, compared with the regression coefficient of −10.63 (95% CI, −20.79 to −0.47) we presented in the article after adjustment for ultrasound-based gestational age. The reason is probably that large random errors of gestational age affect estimates much more than smaller systematic errors. Furthermore, perfluorinated chemicals may not impair early fetal growth.

Birth weight is a function of fetal growth and the duration of the pregnancy, but until better estimates become available, we must use these imprecise measures of gestational age to determine the duration of pregnancy. If the exposure under study slows early fetal growth, adjustment for gestational age based on ultrasound may underestimate an effect of the exposure on fetal growth and overestimate a risk of preterm birth. A similar bias is expected when the results are adjusted for gestational age based on LMP data if the exposure prolongs menstrual cycles.

We thank Slama et al. for reminding us that estimating gestational age is always a problem.
